# PCNA and JNK1‐Stat3 pathways respectively promotes and inhibits diabetes‐associated centrosome amplification by targeting at the ROCK1/14‐3‐3σ complex in human colon cancer HCT116 cells

**DOI:** 10.1002/jcp.27813

**Published:** 2018-11-27

**Authors:** Yu Cheng Lu, Pu Wang, Jie Wang, Ronald Ma, Shao Chin Lee

**Affiliations:** ^1^ School of Life Sciences, Shanxi University Taiyuan Shanxi China; ^2^ Central Laboratory, Linyi People's Hospital Linyi Shandong China; ^3^ School of Acupuncture and Moxibustion, Shanxi University of Traditional Chinese Medicine Taiyuan Shanxi China; ^4^ Department of Medicine and Therapeutics The Chinese University of Hong Kong Shatin Hong Kong SAR China; ^5^ School of Life Sciences, Jiangsu Normal University Xuzhou Jiangsu China

**Keywords:** centrosome amplification, diabetes, JNK1/Stat3, NPM, PCNA, ROCK1/14‐3‐3σ

## Abstract

We have recently reported that type 2 diabetes promotes centrosome amplification via enhancing the expression, biding, and centrosome translocation of rho‐associated coiled‐coil containing protein kinase 1 (ROCK1)/14‐3‐3σ complex in HCT116 cells. In the functional proteomic study, we further investigated the molecular pathways underlying the centrosome amplification using HCT116 cells. We found that treatment of HCT116 cells with high glucose, insulin, and palmitic acid triggered the centrosome amplification and increased the expressions of proliferating cell nuclear antigen (PCNA), nucleophosmin (NPM), and 14‐3‐3σ. Individual knockdown of PCNA, NPM, or 14‐3‐3σ inhibited the centrosome amplification. Knockdown of PCNA inhibited the treatment‐increased expression of ROCK1, whereas knockdown of ROCK1 did not affect the PCNA expression. High glucose, insulin, and palmitic acid also increased the expressions of c‐Jun N‐terminal kinase‐1 (JNK1) and signal transducer and activator of transcription 3 (Stat3), individual knockdown of which upregulated the treatment‐increased expression of 14‐3‐3σ and promoted the centrosome amplification. In contrast, overexpression of JNK1 inhibited the centrosome amplification. Knockdown of Stat3 enhanced the centrosome translocation of 14‐3‐3σ. Moreover, we showed that knockdown of JNK1 inhibited the treatment‐increased expression of Stat3. Knockdown of PCNA, JNK1, or Stat3 did not have an effect on NPM and vice versa. In conclusion, our results suggest that PCNA and JNK1‐Stat3 pathways respectively promotes and feedback inhibits the centrosome amplification by targeting at the ROCK1/14‐3‐3σ complex, and NPM serves as an independent signal for the centrosome amplification.

**Abbreviations**
AKT: Protein kinase B
JNK: c‐Jun N‐terminal kinase
NPM: Nucleophosmin
PCNA: Proliferating cell nuclear antigen
ROCK1: Rho‐associated coiled‐coil containing protein kinase 1
ROS: Reactive oxygen species
Stat3: Signal transducer and activator of transcription 3

## 1 INTRODUCTION

Empty two characters Centrosomes are small nonmembrane organelles composed of two centrioles surrounded by pericentriolar material in mammalian and lower plant cells. They are the microtubule organizing centers, which are important for cell division and the maintenance of genomic stability (Nigg & Raff, 2009). A normal mitotic cell contains two centrosomes oriented on opposite poles of the cell. These centrosomes are responsible for generating bipolar mitotic spindles which promote proper segregation of chromosomes into two equal daughter cells. Each of the daughter cells contains one centrosome (D'Assoro, Lingle, & Salisbury, 2002).

Centrosome amplification, acquisition of more than two centrosomes in a cell, has been described in various types of cancers, including both solid tumors and hematological malignancies (Nigg, 2006). It severely disturbs mitotic process and cytokinesis due to formation of more than two spindle poles, resulting in chromosome instability due to chromosome missegregation. Notably, Basto et al. (2008) reported that centrosomes amplification is sufficient to initiate tumorigenesis in flies. In a transgenic mice model, centrosome amplification is sufficient to initiate spontaneous tumorigenesis (Levine et al., 2017). These observations strongly suggest that there is a cause and consequence relationship between centrosome amplification and tumorigenesis. Moreover, Godinho et al. (2014) have shown that centrosome amplification can promote cancer cell invasion, which may explain the association between centrosome amplification and poor prognosis in cancer patients (Chan, 2011).

Type 2 diabetes is a common disease, which confers an increased risk for all‐site cancer (Giovannucci et al., 2010) and worsens the cancer prognosis (Mills, Bellows, Hoffman, Kelly, & Gagliardi, 2013). In the development of type 2 diabetes (DeFronzo et al., 2015), it is currently recognized that insulin resistance occurs in prediabetic state that shows hyperinsulinemia due to the compensation for reduced insulin sensitivity. Clinical diabetes appears when fasting blood glucose is abnormally elevated. Hyperinsulinemia persists at this state; however, elevated insulin level is already unable to maintain normal fasting blood glucose level in light of insulin resistance. At the later stage of type 2 diabetes, insulin deficiency develops due to the loss of islet beta cells, with persisting hyperglycemia. Continuous hypersecretion of insulin for compensating insulin insensitivity exhausts beta cells, which leads to beta cell death. Elevated level of free fatty acids is noted since the prediabetic state. Hyperinsulinemia, hyperglycemia, and increase in free fatty acids are present over a long period of time in the course of type 2 diabetes. Palmitic acid, the most common saturated free fatty acid, is often used to investigate the effects of free fatty acids, in particular the adverse effects (Mancini et al., 2015).

Recently, we have reported that type 2 diabetes is associated with an increased level of centrosome amplification in peripheral blood mononuclear cells (PBMC). In HCT116 cell line model in vitro, high glucose, insulin, and palmitic acid increase the expression, binding, and centrosomal translocation of rho‐associated coiled‐coil containing protein kinase 1 (ROCK1) and 14‐3‐3σ. These molecular events are also seen in PBMC from patients with type 2 diabetes (Wang et al., 2018). Our results suggest that centrosome amplification is a candidate biological link between type 2 diabetes and cancer development.

At the cellular level, it is believed that there are four major potential mechanisms for centrosome amplification: (1) Multiple centrosome duplication in one cell cycle can occur when a cell division and centrosome duplication cycle become uncoupled and the centrosome duplication cycle continues whereas the cell cycle is stalled (Fukasawa, 2005); (2) the failure of cytokinesis when a cell fails to complete cytokinesis at the end of mitosis and centrosomes remain in a cell rather then enter into daughter cells (Fukasawa, 2005); (3) abnormal centriole splitting, when two G2 phase centrosomes split to form four centrosomes with one centriole (Fukasawa, 2005); (4) the acentriolar centrosome formation, which is the generation of centrosomes that do not contain centrioles (Fukasawa, 2005).

At the molecular level, many genes and proteins have been shown to promote centrosome amplification. In flies and humans, the overexpression of SAK/PLK4 can drive centriole over‐duplication (Holland et al., 2012). Aurora‐A does not deregulate centrosome duplication but gives rise to extra centrosomes through defects in cell division and consequent tetraploidization (Meraldi, Honda, & Nigg, 2002). Overexpression of Aurora‐A, which is frequently amplified in tumors (Lens, Voest, & Medema, 2010), has been shown to override the spindle assembly checkpoint and thereby results in tetraploid cells which are due to cell division defects (Anand, Penrhyn‐Lowe, & Venkitaraman, 2003; Meraldi et al., 2002). The loss of p53 and the deregulated expression of its regulators Mdm2 as well as downstream targets p21^*Cip1*^ and Gadd45 have been linked to centrosome amplification (Meraldi et al., 2002). BRCA1/2 and ATR also affect centrosome numbers in response to DNA damage (Tutt et al., 1999).

Despite that many genes and proteins are shown to promote centrosome amplification, limited amount of data can be found on signal transduction pathway. Arquint and Nigg (2016) reported that STIL and SAS‐6 are downstream signals of Plk4, forming a Plk4‐STIL‐SAS‐6 pathway. The interaction of the pathway initiated centriole duplication, and overexpression of any one of the components of the pathway resulted centrosome amplification (Arquint & Nigg, 2016). Moreover, in breast cancer cells, E2F regulated centrosome amplification in part through Nek2. Another finding is that Cdk4/Nek2 signaling modulated centrosome amplification (Harrison Pitner & Saavedra, 2013; Lee, Moreno, & Saavedra, 2014). The present study further investigated the signals and signal transduction pathways underlying the centrosome amplification by high glucose, insulin, and palmitic acid using functional proteomic analysis in combination with candidate protein characterization.

## 2 MATERIALS AND METHODS

### 2.1 Chemicals, antibodies, and cells

All chemicals were purchased from Sigma (St. Louis, MO). Gama‐tubulin antibody (No. ab27074; mouse antibody) was purchased from Abcam (Cambridge, UK). Rock1 antibody (No. 4035; rabbit antibody) was provided by cell signaling technology (Boston, MA). 14‐3‐3σ antibody (No. PLA0201; rabbit antibody) was purchased from Sigma (St. Louis, MO). Other antibodies were provided by Cell Signaling Technology (Boston, MA). Human colon cancer HCT116 cells were kindly provided by Dr. B. Vogelstein of the Johns Hopkins University School of Medicine. The cell culture medium and reagents were purchased from Gibco (Beijing, China). The palmitic acid stock was conjugated to fatty acid‐free bovine albumin in a 3:1 molar ratio at 37°C for 1 hr before use. Antigamma tubulin antibody was used to detect centrosome by immunofluorescent staining.

### 2.2 Cell culture

HCT116 cells were maintained in the Dulbecco's modified Eagle medium (DMEM; glucose, 5 mM) supplemented with 10% fetal bovine serum, and 1% penicillin‐streptomycin in a humidified incubator with 5% CO_2_ at 37°C. Cell cultures at approximately 70% confluence were used for all experimental treatments. Cells treated for 48 hr were used for quantification of centrosome number and protein distribution in centrosomes. We performed time course assays and the time point was chosen, since centrosome amplification reached a significantly increased level. Cells treated for 30 hr were used for proteomic and western blot analyses. In experiments involving transfection, cells were treated 24 hr after transfection.

### 2.3 Two dimensional gel electrophoresis (2DE)

Two dimensional gel electrophoresis (2‐DE) analyses were performed using immobilized pH gradient (IPG) with a linear pH gradient from 4 to 7 as the first dimensional gels. IPG strips were rehydrated in re‐swelling buffer (8 M urea, 2% CHAPS, 0.5% pharmalyte, 0.2% DTT) for first dimensional electrophoresis in an IPGphor machine (500 v 1 hr, 1,000 v 1 hr, 3,500 v 1 hr, gradient 8,000 v 1 hr, and 24,000 vh). Total cellular protein samples were applied using sample loading cup method. Focused IPG strips were frozen at −20°C till use.

Before the second‐dimensional run, IPG strips were equilibrated with sodium dodecyl sulfate (SDS), after which they were placed on the top of 12% SDS‐polyacrylamide gel electrophoresis (PAGE) (Bio‐Rad PROTEAN II xi cell) and sealed with 0.5% agarose containing trace amount of bromophenol blue for the second‐dimensional separation under 12 mA 2 hr then 24 mA till the bromophenol blue reached the bottom of electrophoresis cell. Peptides separated on 2‐D gels were visualized by silver staining method. Stained gels were scanned using an ImageScanner and analyzed by the software package ImagMaster 2D Platinum. Protein spots of interests were excised with a scalpel for in‐gel tryptic digestion. Briefly, excised gels were destained with 30 mM potassium ferricyanide and 50 mM sodium thiosulfate till colorless, and then washed two times with 25 mM ammonium bicarbonate, air dryed, and overnight digestion at 37°C in 25 mm ammonium bicarbonate containing 10 ng/mL of trypsin.

### 2.4 Protein identification using peptide mass fingerprinting (PMF)

Peptide mass fingerprinting (PMF) involved mass determination of tryptic fragments using MS in combination with mass database matching for peptide identifications. MS was performed using the Applied Biosystems5800 Proteomics Analyzer MALDI‐TOF/TOF (Applied Biosystems, Framingham, MA). Tryptic peptide mixtures (0.5 ml) were spotted on a 192‐well target plate and crystallized with 0.5 ml of α‐Cyano‐4‐hydroxycinnamic acid (CHCA) matrix solution (5 mg/mL). MS data were automatically acquired with a trypsin auto digest exclusion list and the 10 most intense ions selected for MS/MS. The collision gas was nitrogen air and the energy was 1 kV. Interpretation was carried out using the GPS Explorer software and database searching was done using the MASCOT program (Matrix Science, London, UK). Combined MS‐MS/MS search were conducted with the following settings: NCBI database, all entries, peptide tolerance at 200 ppm, MS/MS tolerance at 0.5 Da, carbamidomethylation of cysteine (fixed modification), and methionine oxidation (variable modifications).

### 2.5 Confocal microscopy

A cover slip was placed in a well of a 6‐well plate. HCT116 cells were plated at a density of 50,000 cells per well. Cells grown on the cover slips were fixed in cold methanol and acetone (1:1; v:v) for 6 min at −20°C, followed by three washes with phosphate‐buffered saline (PBS; 10 min each time). Then, the cells were incubated with 0.1% Triton X‐100 for 15 min and 3% bovine serum albumin (BSA) for 1 hr. The cells were incubated with a primary antibody in 3% BSA in PBS overnight at 4°C, washed two times with PBS, and incubated with a FITC‐conjugated secondary antibody in 3% BSA in 1 × PBS for 1 hr at room temperature in the dark. Finally, the cells were mounted with mounting medium. Confocal microscopy was performed using the Zeiss LSM880 microscope (Oberkochen, Germany) with a 1.4 NA oil‐immersion lens, and image processing was performed with Zen software (Oberkochen, Germany).

### 2.6 Western blot analysis

HCT116 cells were lysed in radioimmunoprecipitation assay (RIPA) buffer (150 mM NaCl, 50 mM Tris‐HCl, pH 7.2, 1% Triton X‐100%, and 0.1% SDS). Proteins were separated by polyacrylamide gel electrophoresis and transferred onto polyvinylidene difluoride (PVDF) membrane. After blocking for 1 hr at room temperature with TBST containing 0.05% (v/v) Tween‐20% and 5% (w/v) nonfat milk, the membranes were incubated with primary antibodies overnight at 4°C, followed by washes with TBST containing 0.05% Tween‐20. The membranes were then incubated with a horseradish peroxidase‐conjugated secondary antibody for 1 hr at room temperature. ECL reagents (Thermo Biosciences, MA) were used to visualize the protein bands which were captured on X‐ray film.

### 2.7 Knockdown of protein level

The predesigned small interfering RNA (siRNA) oligonucleotides (Sangon Technology, Shanghai, China) were: 1: c‐Jun N‐terminal kinase‐1 (JNK1), 5′‐GCUCAGGAGCUCAAGGAAUTT‐3′ (sense) and 5′‐AUUCCUUGAGCUCCUGAGCTT‐3′ (antisense); 2: signal transducer and activator of transcription 3 (Stat3), 5′‐GGGACCUGGUGUGAAUUAUTT‐3′ (sense) and 5′‐AUAAUUCACACCAGGUCCCTT‐3′ (antisense); 3: proliferating cell nuclear antigen (PCNA), 5′‐CAGUAUGUCUGCAGAUGUATT‐3′ (sense) and 5′‐UACAUCUGCAGACAUACUGAG‐3′ (antisense); 4: 14‐3‐3σ, 5′‐ACCUGCUCUCAGUAGCCUATT‐3′ (sense) and 5′‐UAGGCUACUGAGAGCAGGUTT‐3′ (antisense); and 5: nucleophosmin (NPM), 5′‐UGAUGAAAAUGGCACCAGTT‐3′ (sense) and 5′‐CUGGUGCUCAUUUUCAUCATT‐3′ (antisense). HCT116 cells (5 × 10^4^ cells per well) were seeded in 6‐well plates and cultured for 24 hr, and then were transfected with 200 pM siRNA oligonucleotides using Lipofectamine^TM^ 2000 transfection reagent (Invitrogen, CA), according to the manufacturer's instructions. The protein level was evaluated by western blot analysis after 24 hr of transfection.

### 2.8 Overexpression of JNK1

pcDNA3.1‐JNK1 was constructed commercially by Sangon Technology, Shanghai, China. Control pcDNA3.1 and pcDNA3.1‐JNK1 respectively were transfected into HCT116 cells in 6‐well plates using Lipofectamine^TM^ 2000 transfection reagent, according to the manufacturer's instructions. After 24 hr, treated group, pcDNA3.1 group, and pcDNA3.1‐JNK1 group cells were replaced with 10% FBS DMEM (Glucose 15 mM, insulin 5 nM, and palmitic acid 150 μM). The protein levels of JNK1 after transfection were determined using western blot analysis.

### 2.9 Statistical analysis

All the experiments were performed in triplicate. The data are expressed as the mean ± SD. Multigroup comparisons were performed using one‐way analysis of variance (ANOVA) analysis. The statistical analysis software package SPSS21 was used for the statistical comparisons. A *p* value < 0.05 was considered to be statistically significant.

## 3 RESULTS

### 3.1 High glucose, insulin, and palmitic acid induce HCT116 cells centrosome amplification

**Figure 1:**
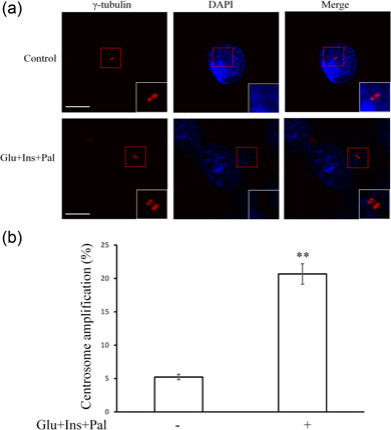
High glucose, insulin, and palmitic acid induce centrosome amplification. (**a**) Image of centrosome amplification; (b) high glucose, insulin, and palmitic acid can promote centrosome amplification in HCT116 cells. Glu: glucose, 15 mM; Ins: insulin, 5 nM; Pal: palmitic acid, 150 μM. **p < 0.01, compared with that in the control group. White scale bar represents 5 μm [Color figure can be viewed at wileyonlinelibrary.com]

We treated HCT116 cells with high glucose (15 mM), insulin (5 nM), and palmitic acid (150 μM) for 48 hr and quantified the centrosomes in each cell. As shown in Figures 1a,b high glucose, insulin, and palmitic acid together were able to induce moderate centrosome amplification of HCT116 cells; under the experimental conditions, most cells with centrosome amplification had 3–6 centrosomes per cells (Figure 1a). In few cells, the number of centrosome could go up to more than 10. The exact number was difficult to determine, since individual centrosome clustered together and were not distinguishable. Experimental treatment increased the level of centrosome amplification events by approximately four folds.

### 3.2 Proteomic analysis reveals nine peptide spots associated with the centrosome amplification

**Figure 2:**
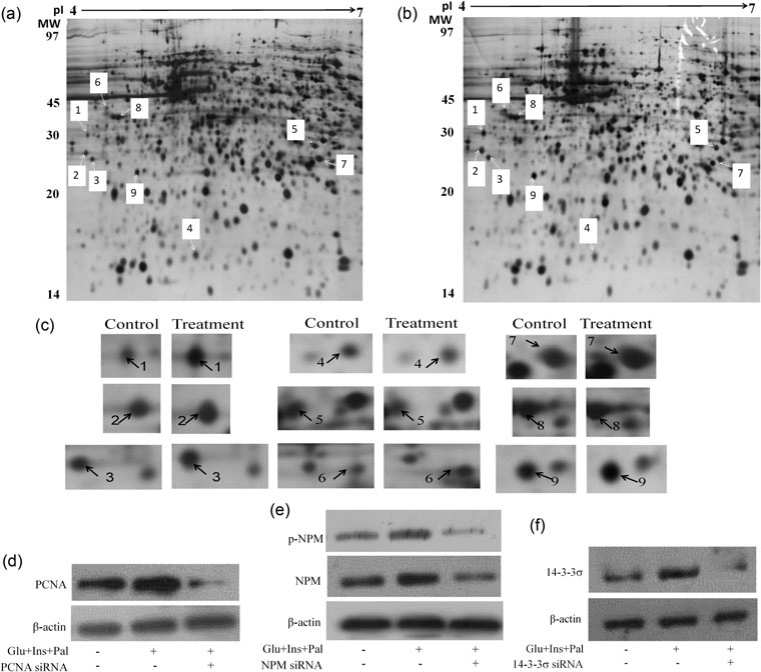
Proteomic analysis identifies nine peptides associated with the centrosome amplification. (a) and (b) Global view of the peptide spots on the two dimensional gels of the control and treated samples, respectively. The differentially expressed spots were labeled. (c) Localized view of the differentially expressed peptide spots on the two dimensional gels. (d–f) Western blot analyses confirmed that the protein levels of PCNA, NPM, and 14‐3‐3σ were upregulated after the treatment, respectively, which was inhibited by their siRNA. Glu: glucose, 15 mM; Ins: insulin, 5 nM; Pal: palmitic acid, 150 μM. NPM: nucleophosmin; PCNA: proliferating cell nuclear antigen; siRNA: small interfering RNA

To examine the molecular basis underlying the centrosome amplification, we performed proteomic analysis to search for proteins responsive to the experimental treatment, which were considered as proteins associated with the centrosome amplification. Protein compositions of control (Figure 2a), treated (Figure 2b) samples were separated on two dimensional gels, and the images of control and treated samples were compared. We found that nine peptide spots were differentially expressed, with two peptide spots downregulated and seven peptide spots upregulated (Figures 2a–c). The identities of these proteins were obtained using the PMF technology (Table 1). The calibrated molecular weight and isoelectric point (pI) values were comparable to the theoretical molecular and pI values of the proteins (Table 1), which supported the identification by PMF.

**Table 1:** Identification of proteins of interests using peptide mass fingerprinting

Spot No.	Protein description	Accession no.	Protein score CI%^a^	MW(kDa)		pI value (pH)		Type of change^b^
				Calibrated	Theoretical	Calibrated	Theoretical	
1	PCNA	gi∣770386238	100	25–30	29	4–5	4.62	+
2	Full = 14‐3‐3 protein sigma	gi∣398953	100	25–30	27	4–5	4.68	+
3	5 Proteasome subunit alpha type‐5	gi∣756142582	100	25–30	25	4–5	4.72	+
4	Cellular retinoic acid‐binding protein 2	gi∣119573307	100	15–20	15	5–6	5.42	−
5	Proteasome subunit, alpha type,1	gi∣119588884	100	25–30	30	6–7	6.51	−
6	The Hcv ires bound to the human ribosome	gi∣887492879	100	30–35	32	4–5	4.79	+
7	Phosphoglycerate mutase 1	gi∣4505753	100	25–30	28	6–7	6.67	+
8	Nucleophosmin	gi∣119581852	99	30–35	31	4–5	4.8	+
9	Rho GDP dissociation inhibitor (GDI) alpha	gi∣119610104	100	20–25	23	4–5	5.02	+

*Note.* PCNA: proliferating cell nuclear antigen.

^a^ Protein score CI% is the confidence interval of identified proteins calculated from MS data.

^b^ (+), protein level was upregulatied; (−), protein level was downregulated; compared with those in the control samples.

Amongst the nine proteins, we were interested in PCNA, NPM, and 14‐3‐3σ, which were chosen for further functional analyses. Western blot analyses confirmed that the expression levels of PCNA, NPM, and 14‐3‐3σ were upregulated by high glucose, insulin, and palmitic acid, which were inhibited by their specific siRNA (Figures 2d‐f). Moreover, phosphorylation of NPM was also upregulated by the experimental treatment (Figure 2e).

### 3.3 PCNA, NPM, and 14‐3‐3σ are signal mediators for the centrosome amplification

**Figure 3:**
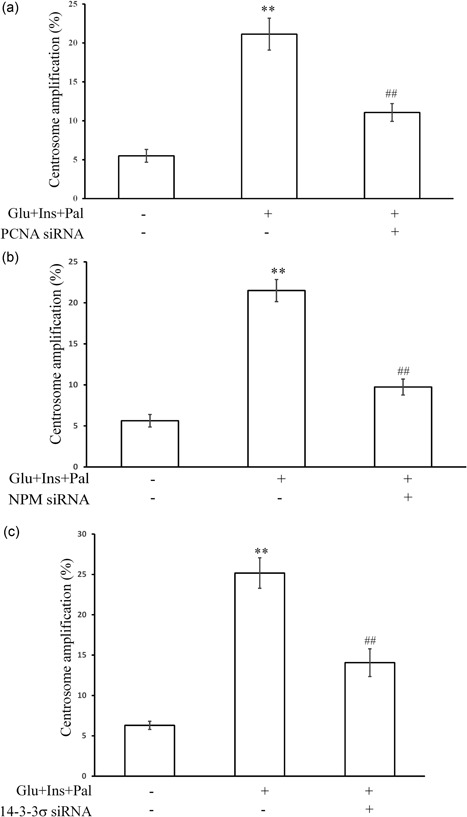
PCNA, NPM, and 14‐3‐3σ mediate the centrosome amplification. (a) PCNA siRNA inhibited the centrosome amplification. (b) NPM siRNA inhibited the centrosome amplification. (c) 14‐3‐3σ siRNA inhibited the centrosome amplification.Glu: glucose, 15 mM; Ins: insulin, 5 nM; Pal: palmitic acid, 150 μM. **p < 0.01, compared with that in the control group; ^##^ *p* < 0.01, compared with that in the samples treated with Glu, Ins, and Pal. NPM: nucleophosmin; PCNA: proliferating cell nuclear antigen; siRNA: small interfering RNA

We next investigated whether PCNA, NPM, and 14‐3‐3σ mediated the centrosome amplification. If they did, individually knockdown of their protein levels using siRNA should inhibit the centrosome amplification. Indeed, as shown in Figures 3a–c respectively, individually knockdown of the expression level of PCNA, NPM, and 14‐3‐3σ inhibited the centrosome amplification.

### 3.4 PCNA targets at ROCK1 to promote the centrosome amplification

**Figure 4:**
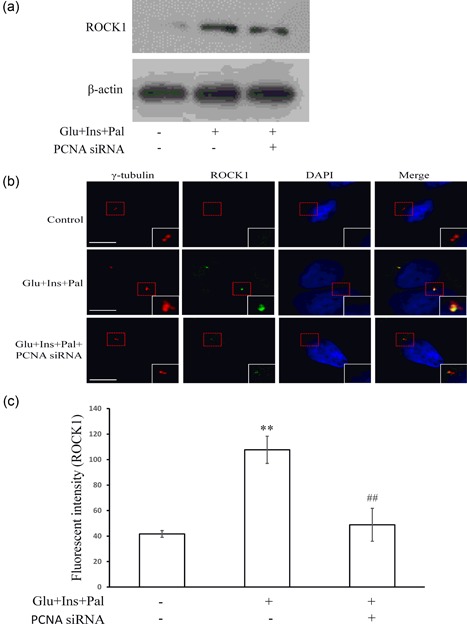
PCNA promotes centrosome amplification by targeting at ROCK1. (a) PCNA siRNA inhibited the protein level of ROCK1. (b) and (c) PCNA siRNA inhibited the centrosome translocation of ROCK1. Glu: glucose, 15 mM; Ins: insulin, 5 nM; Pal: palmitic acid, 150 μM. **p < 0.01, compared with that in the control group; ^##^ *p* < 0.01, compared with that in the samples treated with Glu, Ins and Pal. White scale bar represents 5 μm. PCNA: proliferating cell nuclear antigen; ROCK1: rho‐associated coiled‐coil containing protein kinase 1; siRNA: small interfering RNA [Color figure can be viewed at wileyonlinelibrary.com]

We have recently reported that the ROCK1/14‐3‐3σ complex mediated the centrosome amplification (Wang et al., 2018). In the present study, we were interested to investigate the relationships between ROCK1/14‐3‐3σ complex and PCNA as well as NPM. We found that NPM was not upstream or downstream of others. However, the knockdown of PCNA inhibited the treatment‐increased expression of ROCK1 (Figure 4a) as well as the treatment‐induced translocation of ROCK1 to the centrosomes (Figures 4b,c). In contrast, the knockdown of ROCK1 did not change the expression level of PCNA (Supporting Information Figure S1).

### 3.5 JNK1 and Stat3 inhibit the centrosome amplification

**Figure 5:**
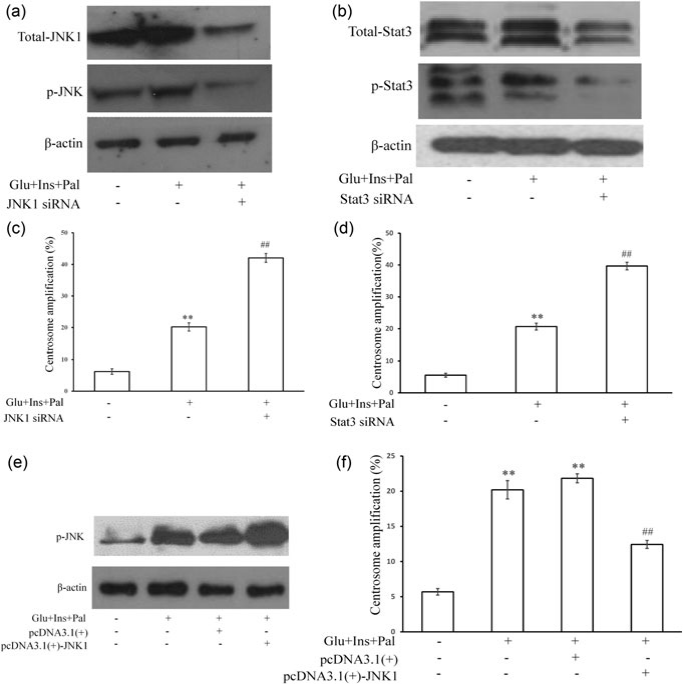
JNK1 and Stat3 pathway can downregulate centrosome amplification in HCT116 cells treatment with high glucose, insulin, and palmitic acid. (a) High glucose, insulin, and palmitic acid increased protein level of total JNK1 and phosphorylated JNK, which was inhibited by JNK1 siRNA. (b) High glucose, insulin, and palmitic acid increased protein level and phosphorylation of Stat3, which was inhibited by Stat3 siRNA. (c) and (d) JNK1 or Stat3 siRNA increased centrosome amplification. (e) Overexpression of JNK1 increased the phosphorylation level of JNK. (f) Centrosome amplification was downregulated by overexpression of JNK1. Glu: glucose, 15 mM; Ins: insulin, 5 nM; Pal: palmitic acid, 150 μM. **p < 0.01, compared with that in the control group; ^##^ *p* < 0.01, compared with that in the samples treated with Glu, Ins, and Pal. JNK1: c‐Jun N‐terminal kinase; siRNA: small interfering RNA; Stat3: signal transducer and activator of transcription 3

In an experiment which examined candidate proteins for centrosome amplification. We found that the expression and activation levels of JNK1 (Figure 5a), Stat3 (Figure 5b) were both upregulated by high glucose, insulin, and palmitic acid, which were inhibited by their specific siRNA (Figures 5a,b). Interestingly, the knockdown of JNK1 (Figure 5c) or Stats (Figure 5d) using their siRNA increased the treatment‐induced centrosome amplification. In contrast, the overexpression of JNK1 (Figure 5e) inhibited the centrosome amplification (Figure 5f). We did not test whether the overexpression of Stat3 could inhibit the centrosome amplification.

### 3.6 JNK1‐Stat3 is the feedback inhibition loop which targets at 14‐3‐3σ

**Figure 6:**
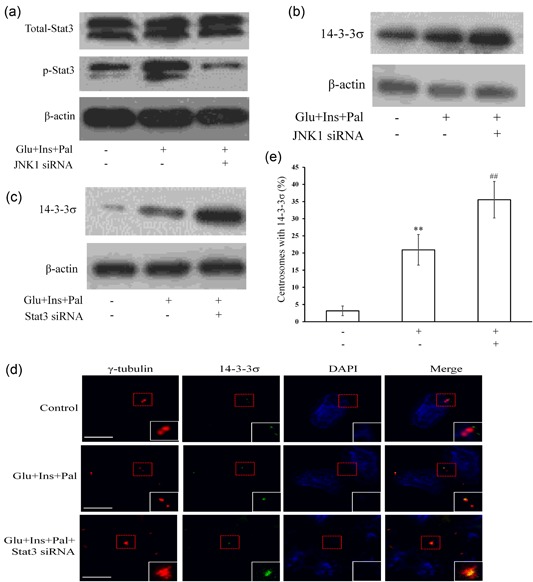
JNK1‐Stat3 is a pathway targeting at 14‐3‐3σ. (a) Treatment‐increased Stat3 phosphorylation was inhibited by JNK1 siRNA. (b) 14‐3‐3σ expression was enhanced by JNK1 siRNA. (c) Stat3 siRNA increases protein level of 14‐3‐3σ. (d) and (e) Stat3 siRNA increased the centrosome translocation of 14‐3‐3σ. Glu: glucose, 15 mM; Ins: insulin, 5 nM; Pal: palmitic acid, 150 μM. **p < 0.01, compared with that in the control group; ^##^ *p* < 0.01, compared with that in the samples treated with Glu, Ins, and Pal. White scale bar represents 5 μm. JNK1: c‐Jun N‐terminal kinase; siRNA: small interfering RNA; Stat3: signal transducer and activator of transcription 3 [Color figure can be viewed at wileyonlinelibrary.com]

Though ROCK1/14‐3‐3σ complex mediated the centrosome amplification (Wang et al., 2018), we further examined the relationships amongst JNK1, Stat3, and the ROCK1/14‐3‐3σ complex. Knockdown of JNK1 inhibited the treatment‐increased p‐Stat3 (Figure 6a). In contrast, the knockdown of Stat3 did not change the expression and activation of JNK1. Moreover, the knockdown of JNK1 (Figure 6b) or Stat3 (Figure 6c) promoted the expression of 14‐3‐3σ. Moreover, the knockdown of Stat3 promoted centrosome translocation of 14‐3‐3σ (Figures 6d,e).

## 4 DISCUSSION

We have recently reported that increased expression and centrosomal translocation of ROCK1/14‐3‐3σ complex mediates centrosome amplification by high glucose, insulin, and palmitic acid (Wang et al., 2018). In the present study, we confirmed that high glucose, insulin, and palmitic acid could induce centrosome amplification (Figures 1a,b) 14–3–3σ is a signal mediator (Figures 2f and 3c), agreeing with our previous report (Wang et al., 2018). Through proteomic analysis, we identified nine proteins associated with the centrosome amplification, which included PCNA, NPM, and 14‐3‐3σ (Figures 2a–c; Table 1). Results from functional assays showed that PCNA targeted at ROCK1 to promote centrosome amplification (Figures 3a and 4a–c). NPM remained to be an independent signal, since knockdown of others did not change its expression or phosphorylation, and knockdown of NPM did not affect other signals (Supporting Information Figure S2).

**Figure 7:**
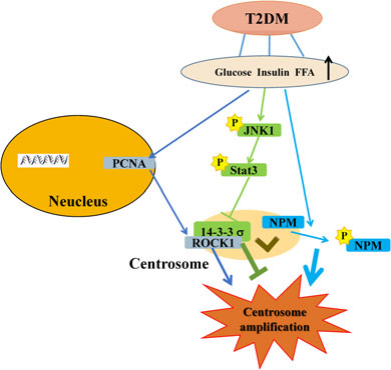
Summary scheme: PCNA–ROCK1, JNK1–Stat3–14‐3‐3σ, and NPM pathways regulate the centrosome amplification triggered by high glucose, insulin, and palmitic acid. The experimental treatment promotes centrosome amplification via PCNA–ROCK1, and NPM pathways and activates a feedback inhibition pathway of JNK1–Stat3–14‐3‐3σ. JNK1: c‐Jun N‐terminal kinase; NPM: nucleophosmin; PCNA: proliferating cell nuclear antigen; ROCK1: rho‐associated coiled‐coil containing protein kinase 1; Stat3: signal transducer and activator of transcription 3 [Color figure can be viewed at wileyonlinelibrary.com]

In addition, results also showed that JNK1 and Stat3 formed a pathway of JNK1–Stat3 (Figure 6a), inhibition of which increased the centrosome amplification (Figures 5c–f). Knockdown of JNK1 or Stat3 enhanced the treatment‐increased 14‐3‐3σ (Figures 6b,c). Knockdown of Stat3 enhanced the treatment‐increased centrosome translocation of 14‐3‐3σ (Figures 6d,e). In contrast, the overexpression of JNK1 inhibited the centrosome amplification. Thus, the experimental treatment activated the JNK1–Stat3 pathway (Figures 5a,b) as a feedback loop that targeted at 14‐3‐3σ in the ROCK1/14‐3‐3σ complex to inhibit the centrosome amplification. Therefore, the experimental treatment activated PCNA–ROCK1 and NPM pathways to promote centrosome amplification. It simultaneously activated a feedback JNK1–Stat3 pathway to inhibit the centrosome amplification (Figure 7). Therefore, it is assumed that the feedback inhibition loop is not activated powerfully enough to counteract the actions of the centrosome amplification‐promoting pathways, thus, centrosome amplification occurs upon the treatment.

PCNA is a protein that is found to be in the nucleus, which functions as a cofactor of DNA polymerase for DNA synthesis (Shiomi & Nishitani, 2017). It strengthens the binding‐a ring‐shaped homotrimer which is of vital importance for the genomic stability, between polymerase and DNA. There is no solid evidence that PCNA and centrosome have a structural or functional relationship. It is found that expression level of Nlp correlates with the expression level of PCNA (Zhao et al., 2012). However, in colorectal cancer, the expression of Aurora members does not correlate with the PCNA expression (Takahashi et al., 2000). Our results showed that PCNA controlled the expression of 14‐3‐3σ and its translocation to the centrosomes (Figures 4a,b) for centrosome amplification. This, for the first time, shows a relationship between PCNA and centrosome, at least in the induction of the centrosome amplification. However, how PCNA affects expression and centrosomal location of 14‐3‐3σ remains unknown.

NPM, also named as B23, localized in granular regions of the nucleolus and has been shown to be associated with preribosomal particles (Okuda et al., 2000). It is identified as one of the targets of CDK2/cyclin E, which can enhance centrosome duplication. Nonphosphorylated NPM binds to centrosomes (Tokuyama, Horn, Kawamura, Tarapore, & Fukasawa, 2001). After Cdk2 and Cyclin E phosphorylate Thr199 in NPM, NPM dissociates from centrosomes, which in turn triggers centrosome duplication (Grisendi et al., 2005). NPM loss leads to unrestricted centrosome duplication and genomic instability in mouse embryonic fibroblasts (Xia et al., 2013). In the present experiment, we found that the expression and phosphorylation levels of NPM were upregulated by the treatment of high glucose, insulin, and palmitic acid, which concomitantly occurred with enhanced centrosome amplification (Figures 2e and 3b). Knockdown of NPM using siRNA inhibited the treatment‐induced upregulation of NPM expression, NPM phosphorylation, and centrosome amplification (Figure 2e). Our results suggest that NPM mediates centrosome amplification, which is in agreement with the observations by others (Grisendi et al., 2005).

The c‐Jun N‐terminal kinases, a member of mitogen‐activated protein kinase family of serine/threonine kinases, are ubiquitously expressed intracellular signaling molecules that are activated by cytokines and exposure to environmental stresses such as osmotic stress, redox stress, and radiation (Kyriakis & Avruch, 2001). More recent experimental data have shown that JNK signaling is involved in the regulation of cellular processes, including apoptosis and survival signaling, T‐cell maturation, brain development, cardiac hypertrophy, and cell cycle (Davis, 2000). Little is known about the role of JNK in centrosome homeostasis and, more specifically, centrosome amplification.

However, there are correlates with JNK and centrosome. Inhibition of centrosomal JNK by HSP70 contributes to the repair of heat shock‐induced damage to centrosomes (Brown, Hong‐Brown, Doxsey, & Welch, 1996). Apoptosis of acentrosomal cells is mediated by JNK signaling, which also drives compensatory proliferation to maintain tissue integrity and viability (Poulton, Cuningham, & Peifer, 2014). In addition, the guts irradiated with 2Gy shows increased JNK and AKT activities as well as centrosome amplification in intestinal stem cells of adult midgets (Pyo et al., 2014). In the present study, we found that JNK1 is activated during treatment‐induced centrosome amplification (Figures 5a,c). Inhibition and overexpression of JNK1 promotes and inhibits the centrosome amplification, respectively (Figures 5e,f). Thus, JNK1 inhibits centrosome amplification, at least under the experimental conditions. The treatment activates JNK1 as a stress response to protect cells for normal function. This is the first time to show a role of JNK1 in the control of centrosome amplification.

Obesity, a worldwide epidemic, is a type of health impairment resulting from aberrant or excessive adipose accumulation (McClean, Kee, Young, & Elborn, 2008). There is an association between type 2 diabetes and obesity; a significant proportion of patients with type 2 diabetes have increased body mass index (Lee et al., 2018). Adipose tissue is composed of adipocytes and a vascular‐stromal fraction, which contains macrophages, fibroblasts, endothelial cells, and pre‐adipocytes. In addition to the regulation of whole‐body fatty acid homeostasis, adipose tissue functions as a complex endocrine organ, secreting adipokines, such as leptin, adiponectin, visfatin, tumor necrosis factor‐α, interleukin‐6, monocyte chemotactic protein‐1, and adipocyte fatty acid‐binding protein (Hsu, Wu, Chang, & Lin, 2015). Leptin induces matrix metalloproteinase 7 expression to promote ovarian cancer cell invasion by activating ERK and JNK pathways (Ghasemi, Hashemy, Aghaei, & Panjehpour, 2018). Genistein inhibited JNK activation, which inhibited the TNF‐α‐mediated downregulation of adiponectin (Yanagisawa et al., 2012). Visfatin may represent a pro‐inflammatory cytokine that is influenced by insulin/insulin sensitivity via the NF‐κB and JNK pathways (McGee et al., 2011). Although these adipokines are associated with JNK activity or signaling pathways, whether these adipokines can alter the diabetes‐associated centrosome amplification remains to be further studied, in the light the JNK was found to inhibit the centrosome amplification in the study (Figure 5f).

Signal transducer and activator of transcription 3, a member of the STAT protein family, is a transcription factor which in humans is encoded by the *STAT* gene (Akira et al., 1994). Stat3 mediates the expression of a variety of genes in response to cell stimuli, and thus plays a key role in many cellular processes such as cell growth and apoptosis (Yuan et al., 2004). A recent study suggests that Stat3 is also involved in centrosome duplication. For example, Metge et al. reported that Stat3 apparently regulates γ‐tubulin levels (Metge, Ofori‐Acquah, Stevens, & Balczon, 2004). Furthermore, Morris et al. reported that Stat3/Stathmin/PLK1 can regulate centrosomal γ‐tubulin levels which allowed centrosome to position themselves together, which result centrosome clustering and bipolar spindle in cancer cells (Morris et al., 2017). In the present study, we found that activation of Stat3 inhibited the centrosome amplification by high glucose, insulin and palmitic acid (Figures 5b,f) which clearly shows that Stat3 can negatively regulate centrosome amplification.

Results from existing studies suggest that a crosstalk and the balance between MAPK and JAK/STAT pathway might be involved in T‐2 toxin‐induced apoptosis inRAW264.7 cells (Wu, Wang, Wan, Li, & Yuan, 2014). JNK, as an upstream signal, has opposite effects on Stat3 phosphorylation. Activation of JNK can upregulate of serine phosphorylation and downregulate tyrosine phosphorylation of Stat3 (Gkouveris, Nikitakis, Karanikou, Rassidakis, & Sklavounou, 2016). Anisomycin triggered apoptosis by JNK1/2/AP‐1/STAT1/STAT3/Bim/Bcl‐xL/Bax/Bak signaling in Jurkat cells (Zhou et al., 2016). Our results showed that JNK1 is upstream of Stat3, and, for the first time, have shown that JNK1‐Stat3 pathway inhibits centrosome amplification (Figure 6a), which targets at 14‐3‐3σ (Figures 6b–e) in the ROCK1/14‐3‐3σ complex.

In our recent report (Wang et al., 2018), we showed that the protein levels of ROCK1 and 14‐3‐3σ as well as centrosome amplification were all increased in PBMC from the patients with type 2 diabetes. In HCT116 cells treated with high glucose, insulin, and palmitic acid, the protein levels of ROCK1 and 14‐3‐3σ were as well as centrosome amplification were also all upregulated. Knockdown of ROCK1 or 14‐3‐3σ attenuated the treatment‐elicited centrosome amplification. The results suggest that type 2 diabetes promotes centrosome amplification via ROCK1 and 14‐3‐3σ, with high glucose, insulin, and palmitic acid as the triggers.

However, the in vivo relevance of the pathways discovered in vitro in the present study requires verification under in vivo setting. We are investigating the protein levels of PCNA and p‐NPM in the PBMC samples from healthy subjects and those with type 2 diabetes, to obtain evidence that can link PCNA and NPM to centrosome amplification in type 2 diabetes (Wang et al., 2018). Similarly, we are building a diabetic mice model using streptozotocin, in an attempt for correlating the levels centrosome amplification with PCNA and p‐NPM. It is difficult to assess the role of JNK1 and Stat3 in the diabetes‐associated centrosome amplification in vivo. Perhaps, genetically modified animal models (i.e., knockout or overexpression) are useful, which is beyond our ability. Importantly, we found that when we treated IEC‐6 rat colon epithelial cells, which are immortalized but noncancerous cells, with high glucose, insulin, and palmitic acid, the levels PCNA, p‐NPM, ROCK1, and 14‐3‐3σ as well as centrosome amplification were all upregulated significantly (Lu et al. unpublished data). We are designing protocols to inoculate the IEC‐6 cell with or without treatment‐increased centrosome amplification into nude mice, for more direct evidence for a cause and consequence relationship amongst the activation of the signaling pathways, centrosome amplification and tumorigenesis.

Discovery of the molecular pathways for the type 2 diabetes‐associated centrosome will be beneficial for development of methods for inhibiting increased centrosome amplification in type 2 diabetes (Wang et al., 2018), that is, by inhibiting the centrosome amplification‐promoting pathways and/or by activating the feedback inhibition loop. It remains to be explored whether the increased centrosome amplification in type 2 diabetes (Wang et al., 2018) contributes to cancer development. If so, inhibition of centrosome amplification in type 2 diabetes would be meaningful for cancer prevention for patients with type 2 diabetes.

## 5 CONCLUSIONS

In the present study, we have identified two novel signal transduction pathways that regulate the centrosome amplification by high glucose, insulin, and palmitic acid, which target at ROCK1/14‐3‐3σ complex in the centrosome. PCNA–ROCK1 pathway promotes the centrosome amplification, whereas JNK1–Stat3–14‐3‐3σ feedback inhibits the centrosome amplification. NPM serves as an independent promoting pathway (Figure 7).

## CONFLICTS OF INTEREST

The authors declare that there are no conflicts of interest.

## Supporting information

Supporting information
